# Dilithium manganese(II) *catena*-tetrakis(polyphosphate), Li_2_Mn(PO_3_)_4_

**DOI:** 10.1107/S1600536813032388

**Published:** 2013-12-04

**Authors:** Meryem Moutataouia, Mohammed Lamire, Mohamed Saadi, Lahcen El Ammari

**Affiliations:** a Laboratoire de Physico-Chimie des Matériaux Inorganiques, Faculté des Sciences Aïn Chock, Casablanca, Morocco; b Laboratoire de Chimie du Solide Appliquée, Faculté des Sciences, Université Mohammed V-Agdal, Avenue Ibn Batouta, BP 1014, Rabat, Morocco

## Abstract

The poly-phosphate Li_2_Mn(PO_3_)_4_ was synthesized and its structure characterized from powder diffraction data by Averbuch-Pouchot & Durif [*J. Appl. Cryst.* (1972), **5**, 307–308]. These authors showed that the structure of this phosphate is isotypic to that of Li_2_Cd(PO_3_)_4_, as confirmed by the present work. The structure is built from infinite zigzag polyphosphate chains, [(PO_3_)^−^]_*n*_, extending along [010]. These polyphosphate chains are connected by sharing vertices with MnO_6_ octa­hedra (site symmetry .*m*.) and Li_2_O_7_ polyhedra, which form also chains parallel to [010]. Adjacent chains are linked by common vertices of polyhedra in such a way as to form porous layers parallel to (100). The three-dimensional framework delimits empty channels extending along [010].

## Related literature   

For potential applications of lithium and manganese phosphates, see: Parada *et al.* (2003 ▶); Jouini *et al.* (2003 ▶); Bian *et al.* (2003 ▶); Aravindan *et al.* (2013 ▶); Drezen *et al.* (2007 ▶); Bakenov & Taniguchi (2010 ▶); Adam *et al.* (2008 ▶). For a previous structure determination from powder data, see: Averbuch-Pouchot & Durif (1972 ▶). For the isotypic structure of Li_2_Cd(PO_3_)_4_, see: Averbuch-Pouchot *et al.* (1976 ▶).

## Experimental   

### 

#### Crystal data  

Li_2_Mn(PO_3_)_4_
*M* *_r_* = 384.70
Orthorhombic, 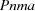

*a* = 9.4295 (2) Å
*b* = 9.2755 (2) Å
*c* = 10.0972 (2) Å
*V* = 883.13 (3) Å^3^
*Z* = 4
Mo *K*α radiation
μ = 2.29 mm^−1^
*T* = 296 K
0.23 × 0.16 × 0.13 mm

#### Data collection  

Bruker X8 APEX diffractometer
Absorption correction: multi-scan (*SADABS*; Sheldrick, 2008 ▶) *T*_min_ = 0.651, *T*_max_ = 0.743
13605 measured reflections
2520 independent reflections
2318 reflections with *I* > 2σ(*I*)
*R*_int_ = 0.024

#### Refinement  

*R*[*F* ^2^ > 2σ(*F* ^2^)] = 0.018
*wR*(*F* ^2^) = 0.051
*S* = 1.09
2520 reflections
97 parameters
Δρ_max_ = 0.52 e Å^−3^
Δρ_min_ = −0.51 e Å^−3^

### 

Data collection: *APEX2* (Bruker, 2009 ▶); cell refinement: *SAINT* (Bruker, 2009 ▶); data reduction: *SAINT*; program(s) used to solve structure: *SHELXS97* (Sheldrick, 2008 ▶); program(s) used to refine structure: *SHELXL97* (Sheldrick, 2008 ▶); molecular graphics: *ORTEP-3 for Windows* (Farrugia, 2012 ▶) and *DIAMOND* (Brandenburg, 2006 ▶); software used to prepare material for publication: *publCIF* (Westrip, 2010 ▶).

## Supplementary Material

Crystal structure: contains datablock(s) I. DOI: 10.1107/S1600536813032388/br2233sup1.cif

Structure factors: contains datablock(s) I. DOI: 10.1107/S1600536813032388/br2233Isup2.hkl

Additional supporting information:  crystallographic information; 3D view; checkCIF report

## Figures and Tables

**Fig. 1.**
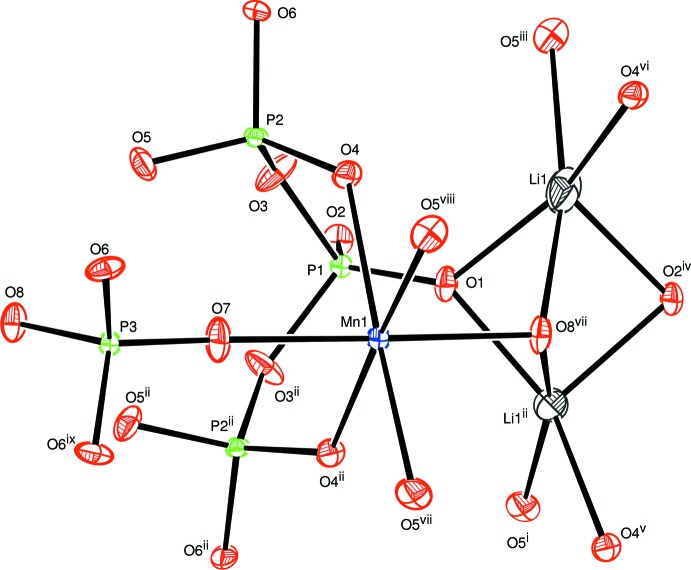
Plot of Li2Mn(PO3)4 crystal structure showing polyhedra linkage. Displacement ellipsoids are drawn at the 50% probability level. Symmetry codes:(i) x - 1/2, y, -z + 3/2; (ii) x - 1/2, -y + 1/2, -z + 3/2; (iii) x, -y + 1/2, z; (iv) -x + 1/2, y + 1/2, z + 1/2; (v) -x + 1/2, -y, z + 1/2; (vi) x - 1/2, y, -z + 1/2; (vii) -x, -y, -z + 1; (viii) -x + 1/2, -y, z - 1/2.

**Fig. 2.**
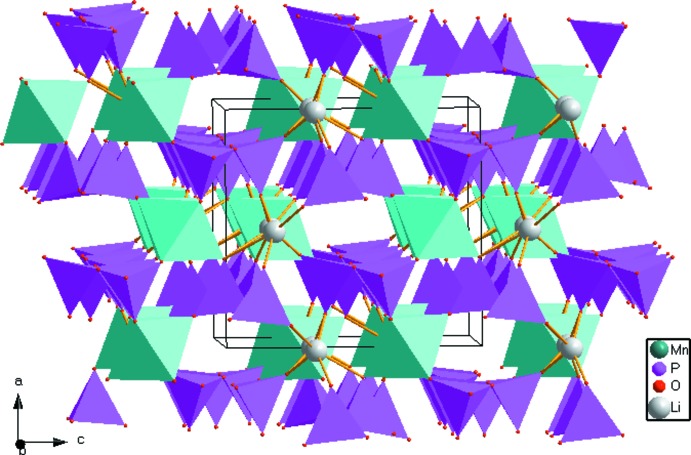
Three-dimensional views of the Li2Mn(PO3)4 framework structure showing emptly tunnels running along b.

## supplementary crystallographic information

### 1. Comment

Due to their interesting physical properties, the lithium and manganese phosphates have a wide domain of applications (Parada *et al.*, 2003; Jouini *et al.*, 2003; Bian *et al.*, 2003). Among these mixed phosphates, the LiMnPO_4_ monophosphate is the most studied, followed by the Li_2_MnP_2_O_7_ diphosphate and the Li_2_Mn(PO_3_)_4_ polyphosphate, which is the object of this work. These phosphate materials are being extensively studied as lithium-ion battery electrodes (Aravindan *et al.*, 2013; Drezen *et al.*, 2007; Bakenov & Taniguchi, 2010; Adam *et al.*, 2008).

Averbuch-Pouchot & Durif (1972) have synthesized the powder of the polyphosphate Li_2_Mn(PO_3_)_4_ and have shown that the structure of this phosphate is isotype to that of Li_2_Cd(PO_3_)_4_ (Averbuch-Pouchot *et al.*, 1976). The present paper describes the crystal structure of the title compound from single-crystal X-ray diffraction data.

The partial three-dimensional plot in Fig.1 illustrates the connection ion-oxygen polyhedra in the crystal structure of the title compound. The phosphorous atoms have a tetrahedral environment with P–O distances varying between 1.4650 (9) Å and 1.5932 (7) Å and the angles O–P–O are in the range of 95.22 (5)–119.93 (5) °. These value are within the limits generally observed in the crystal chemistry of condensed phosphate. The Mn2+ cation is surrounded by a roughly octahedral arrangement of six oxygen atoms and share one edge with Li_2_O_7_ polyhedron in which each Li is coordinated to five oxygen atoms.

The structure of Li_2_Mn(PO_3_)_4_ consists of edge-sharing [MnO_6_] octahedra and [Li_2_O_7_] polyhedra forming an infinite linear chains [Mn–Li–Li–Mn] running parallel to [100], as shown in Fig.2. The (PO_3_)^-^n polyphosphate form also infinite zigzag chains propaging along *b* axis. Adjacent chains are linked together by common vertices of polyhedra in such a way as to form porous layers parallel to (100). The resulting 3-D framework presents empty tunnels running along [010] directions (Fig.2).

### 2. Experimental

The synthesis of the polyphosphate Li_2_MnP_4_O_12_ by wet process, was made starting from the stoechiometric proportions of (LiNO_3_ 99,9%) (I); (Mn(NO_3_)_2_,4H_2_O 99%) (II) and ((NH_4_)_2_HPO_4_ 99%) (III). The starting reagents were made in distilled water solution. A drop by drop of the solution (II) was added on the solution (I), under mechanical agitation, and thereafter the solution (III). The mixture is carried to 373 K until total evaporation of the solution. The residue thus obtained was heated in air, intersected with grindings, until a final temperature of 773 K during 4 h. The final products are of violet colour.

The previous powder of the Li_2_MnP_4_O_12_ phase synthesized by wet process introduced into a platinum crucible, then carried gradually heated at a temperature higher than its melting point (973 K) during 2 h, followed-up by a slow cooling about 5 °K per hour until 773 K. Then, the power supply of the furnace is cut, and cooling is continued until the ambient temperature. The single crystals obtained are of violet colour.

### 3. Refinement

The highest peak and the deepest hole in the final Fourier map are at 0.51 Å and 0.97 Å, from O3 and P1, respectively. The not significant bonds and angles were removed from the CIF file.

### Crystal data

**Table:**

Li_2_Mn(PO_3_)_4_	*F*(000) = 748
*M**_r_* = 384.70	*D*_x_ = 2.893 Mg m^−^^3^
Orthorhombic, *P**n**m**a*	Mo *K*α radiation, λ = 0.71073 Å
Hall symbol: -P 2ac 2n	Cell parameters from 2520 reflections
*a* = 9.4295 (2) Å	θ = 3.0–38.1°
*b* = 9.2755 (2) Å	µ = 2.29 mm^−^^1^
*c* = 10.0972 (2) Å	*T* = 296 K
*V* = 883.13 (3) Å^3^	Block, violet
*Z* = 4	0.23 × 0.16 × 0.13 mm

### Data collection

**Table:**

Bruker X8 APEX diffractometer	2520 independent reflections
Radiation source: fine-focus sealed tube	2318 reflections with *I* > 2σ(*I*)
Graphite monochromator	*R*_int_ = 0.024
φ and ω scans	θ_max_ = 38.1°, θ_min_ = 3.0°
Absorption correction: multi-scan (*SADABS*; Sheldrick, 2008)	*h* = −16→13
*T*_min_ = 0.651, *T*_max_ = 0.743	*k* = −16→13
13605 measured reflections	*l* = −11→17

### Refinement

**Table:**

Refinement on *F*^2^	0 restraints
Least-squares matrix: full	Primary atom site location: structure-invariant direct methods
*R*[*F*^2^ > 2σ(*F*^2^)] = 0.018	Secondary atom site location: difference Fourier map
*wR*(*F*^2^) = 0.051	*w* = 1/[σ^2^(*F*_o_^2^) + (0.0262*P*)^2^ + 0.2295*P*] where *P* = (*F*_o_^2^ + 2*F*_c_^2^)/3
*S* = 1.09	(Δ/σ)_max_ = 0.001
2520 reflections	Δρ_max_ = 0.52 e Å^−^^3^
97 parameters	Δρ_min_ = −0.51 e Å^−^^3^

### Special details

**Table:**

Geometry. All e.s.d.'s (except the e.s.d. in the dihedral angle between two l.s. planes) are estimated using the full covariance matrix. The cell e.s.d.'s are taken into account individually in the estimation of e.s.d.'s in distances, angles and torsion angles; correlations between e.s.d.'s in cell parameters are only used when they are defined by crystal symmetry. An approximate (isotropic) treatment of cell e.s.d.'s is used for estimating e.s.d.'s involving l.s. planes.
Refinement. Refinement of *F*^2^ against all reflections. The weighted *R*-factor *wR* and goodness of fit *S* are based on *F*^2^, conventional *R*-factors *R* are based on *F*, with *F* set to zero for negative *F*^2^. The threshold expression of *F*^2^ > σ(*F*^2^) is used only for calculating *R*-factors(gt) *etc*. and is not relevant to the choice of reflections for refinement. *R*-factors based on *F*^2^ are statistically about twice as large as those based on *F*, and *R*- factors based on all data will be even larger.

### Fractional atomic coordinates and isotropic or equivalent isotropic displacement parameters (Å^2^)

**Table:**

	*x*	*y*	*z*	*U*_iso_*/*U*_eq_	
Mn1	0.012320 (17)	0.2500	0.697042 (16)	0.00824 (4)	
P1	0.30533 (3)	0.2500	0.39321 (3)	0.00708 (5)	
P2	0.29220 (2)	0.03744 (2)	0.610286 (19)	0.00742 (4)	
P3	0.22718 (3)	0.2500	0.98429 (3)	0.00661 (5)	
O1	0.14967 (9)	0.2500	0.37467 (9)	0.01356 (15)	
O2	0.40037 (9)	0.2500	0.27630 (8)	0.01232 (14)	
O3	0.35189 (7)	0.11634 (8)	0.48227 (7)	0.01946 (13)	
O4	0.13548 (6)	0.05951 (7)	0.62027 (6)	0.01146 (10)	
O5	0.38358 (7)	0.07235 (7)	0.72611 (7)	0.01537 (11)	
O6	0.32753 (6)	−0.12374 (7)	0.57702 (7)	0.01270 (10)	
O7	0.14661 (10)	0.2500	0.86024 (9)	0.01801 (18)	
O8	0.38557 (9)	0.2500	0.98024 (8)	0.01291 (15)	
Li1	0.0036 (2)	0.1031 (3)	0.3305 (3)	0.0319 (5)	

### Atomic displacement parameters (Å^2^)

**Table:**

	*U*^11^	*U*^22^	*U*^33^	*U*^12^	*U*^13^	*U*^23^
Mn1	0.00776 (6)	0.00889 (7)	0.00807 (7)	0.000	−0.00102 (5)	0.000
P1	0.00667 (10)	0.00812 (11)	0.00646 (10)	0.000	0.00036 (7)	0.000
P2	0.00715 (7)	0.00576 (8)	0.00935 (8)	−0.00010 (5)	0.00026 (5)	−0.00046 (6)
P3	0.00626 (10)	0.00710 (11)	0.00645 (10)	0.000	−0.00036 (7)	0.000
O1	0.0071 (3)	0.0192 (4)	0.0144 (3)	0.000	−0.0017 (3)	0.000
O2	0.0121 (3)	0.0163 (4)	0.0086 (3)	0.000	0.0034 (3)	0.000
O3	0.0135 (2)	0.0205 (3)	0.0243 (3)	0.0064 (2)	0.0071 (2)	0.0153 (3)
O4	0.0076 (2)	0.0110 (2)	0.0158 (2)	0.00088 (18)	0.00164 (17)	−0.0008 (2)
O5	0.0134 (2)	0.0165 (3)	0.0162 (3)	−0.0019 (2)	−0.0040 (2)	−0.0070 (2)
O6	0.0125 (2)	0.0068 (2)	0.0188 (3)	0.00120 (18)	−0.0044 (2)	−0.0039 (2)
O7	0.0158 (4)	0.0279 (5)	0.0103 (3)	0.000	−0.0058 (3)	0.000
O8	0.0065 (3)	0.0222 (4)	0.0099 (3)	0.000	0.0015 (2)	0.000
Li1	0.0186 (8)	0.0241 (10)	0.0530 (14)	−0.0087 (7)	−0.0128 (8)	0.0163 (10)

### Geometric parameters (Å, º)

**Table:**

Mn1—O7	2.0782 (9)	P2—O3	1.5885 (7)
Mn1—O8^i^	2.1523 (8)	P3—O7	1.4650 (9)
Mn1—O5^i^	2.1888 (6)	P3—O8	1.4941 (8)
Mn1—O5^ii^	2.1888 (6)	P3—O6^iv^	1.5857 (6)
Mn1—O4^iii^	2.2519 (6)	P3—O6^v^	1.5857 (6)
Mn1—O4	2.2519 (6)	Li1—O1	1.988 (2)
P1—O1	1.4797 (9)	Li1—O2^vi^	1.992 (2)
P1—O2	1.4821 (9)	Li1—O4^vii^	2.060 (2)
P1—O3^iii^	1.5932 (7)	Li1—O5^viii^	2.211 (3)
P1—O3	1.5932 (7)	Li1—O8^i^	2.598 (3)
P2—O5	1.4883 (6)	Li1—Li1^iii^	2.725 (5)
P2—O4	1.4953 (6)	Li1—Mn1^vii^	3.290 (2)
P2—O6	1.5681 (6)		
			
O7—Mn1—O8^i^	176.19 (4)	O5—P2—O6	104.64 (4)
O7—Mn1—O5^i^	93.26 (3)	O4—P2—O6	110.79 (4)
O8^i^—Mn1—O5^i^	89.25 (2)	O5—P2—O3	109.51 (4)
O7—Mn1—O5^ii^	93.26 (3)	O4—P2—O3	110.00 (4)
O8^i^—Mn1—O5^ii^	89.25 (2)	O6—P2—O3	100.93 (4)
O5^i^—Mn1—O5^ii^	97.67 (4)	O7—P3—O8	119.67 (5)
O7—Mn1—O4^iii^	87.63 (2)	O7—P3—O6^iv^	109.64 (4)
O8^i^—Mn1—O4^iii^	90.01 (2)	O8—P3—O6^iv^	109.97 (3)
O5^i^—Mn1—O4^iii^	177.06 (2)	O7—P3—O6^v^	109.64 (4)
O5^ii^—Mn1—O4^iii^	79.48 (2)	O8—P3—O6^v^	109.97 (3)
O7—Mn1—O4	87.63 (2)	O6^iv^—P3—O6^v^	95.22 (5)
O8^i^—Mn1—O4	90.01 (2)	O1—Li1—O2^vi^	89.50 (9)
O5^i^—Mn1—O4	79.48 (2)	O1—Li1—O4^vii^	152.74 (16)
O5^ii^—Mn1—O4	177.06 (2)	O2^vi^—Li1—O4^vii^	108.68 (9)
O4^iii^—Mn1—O4	103.37 (3)	O1—Li1—O5^viii^	106.16 (10)
O1—P1—O2	119.93 (5)	O2^vi^—Li1—O5^viii^	118.72 (14)
O1—P1—O3^iii^	110.17 (3)	O4^vii^—Li1—O5^viii^	83.24 (9)
O2—P1—O3^iii^	106.43 (3)	O1—Li1—O8^i^	76.84 (10)
O1—P1—O3	110.17 (3)	O2^vi^—Li1—O8^i^	80.21 (9)
O2—P1—O3	106.43 (3)	O4^vii^—Li1—O8^i^	86.20 (9)
O3^iii^—P1—O3	102.18 (6)	O5^viii^—Li1—O8^i^	160.51 (11)
O5—P2—O4	119.30 (4)		

Symmetry codes: (i) *x*−1/2, *y*, −*z*+3/2; (ii) *x*−1/2, −*y*+1/2, −*z*+3/2; (iii) *x*, −*y*+1/2, *z*; (iv) −*x*+1/2, *y*+1/2, *z*+1/2; (v) −*x*+1/2, −*y*, *z*+1/2; (vi) *x*−1/2, *y*, −*z*+1/2; (vii) −*x*, −*y*, −*z*+1; (viii) −*x*+1/2, −*y*, *z*−1/2.
